# Preferential Cochleotoxicity of Cisplatin

**DOI:** 10.3389/fnins.2021.695268

**Published:** 2021-07-26

**Authors:** Pattarawadee Prayuenyong, David M. Baguley, Corné J. Kros, Peter S. Steyger

**Affiliations:** ^1^Department of Otorhinolaryngology, Head and Neck Surgery, Faculty of Medicine, Prince of Songkla University, Songkhla, Thailand; ^2^Hearing Sciences, Division of Clinical Neurosciences, School of Medicine, University of Nottingham, Nottingham, United Kingdom; ^3^National Institute for Health Research (NIHR) Nottingham Biomedical Research Centre, Nottingham, United Kingdom; ^4^Nottingham Audiology Services, Nottingham University Hospitals NHS Trust, Nottingham, United Kingdom; ^5^School of Life Sciences, University of Sussex, Brighton, United Kingdom; ^6^Translational Hearing Center, Biomedical Sciences, Creighton University, Omaha, NE, United States

**Keywords:** cisplatin, ototoxicity, cochlea, vestibular, cochleotoxicity, vestibulotoxicity

## Abstract

Cisplatin-induced ototoxicity in humans is more predominant in the cochlea than in the vestibule. Neither definite nor substantial vestibular dysfunction after cisplatin treatment has been consistently reported in the current literature. Inner ear hair cells seem to have intrinsic characteristics that make them susceptible to direct exposure to cisplatin. The existing literature suggests, however, that cisplatin might have different patterns of drug trafficking across the blood-labyrinth-barrier, or different degrees of cisplatin uptake to the hair cells in the cochlear and vestibular compartments. This review proposes an explanation for the preferential cochleotoxicity of cisplatin based on current evidence as well as the anatomy and physiology of the inner ear. The endocochlear potential, generated by the stria vascularis, acting as the driving force for hair cell mechanoelectrical transduction might also augment cisplatin entry into cochlear hair cells. Better understanding of the stria vascularis might shed new light on cochleotoxic mechanisms and inform the development of otoprotective interventions to moderate cisplatin associated ototoxicity.

## Introduction

Cisplatin (platinum-based chemotherapy), is the mainstay treatment for curative care of various cancers (Makovec, 2019). Ototoxicity is a common side effect of cisplatin which can limit its usage and dosage (Qi et al., 2019). Ototoxicity refers to drug-induced damage affecting the inner ear structures and related neural tissues, causing cochlear dysfunction (such as hearing loss or tinnitus) and/or vestibular dysfunction (such as vertigo, dizziness, or imbalance) or both (Lanvers-Kaminsky et al., 2017). The damage can manifest in cellular degeneration and/or functional impairment. Unlike other side effects of cisplatin (such as nephrotoxicity), ototoxicity can cause irreversible injury to the inner ear as human inner ear sensory hair cells generally cannot regenerate (Rubel et al., 2013). Furthermore, no known effective protective or curative strategies are presently available for cisplatin-induced ototoxicity although clinical trial data is emerging (Mukherjea et al., 2020).

This review gleans the current evidence of cisplatin-induced ototoxicity and the mechanisms in the light of existing literature and data. Possible mechanisms are suggested as to why cisplatin predominantly affects the cochlea while relatively sparing the vestibular counterpart based on available preclinical and clinical data.

## Similarities and Differences of Cochlear and Vestibular Organs

The cochlear (responsible for hearing) and vestibular (responsible for balance) parts of the inner ear have a very close anatomical relationship, and share a common source of blood and fluid supplies (Flint et al., 2014). These two components have replete similarities in anatomy, cellular and molecular components. The perilymph and endolymph of both structures have similar electrolyte compositions—the perilymph containing low potassium (K^+^) and high sodium (Na^+^) concentrations, and the endolymph containing high K^+^ and low Na^+^ concentrations. Specific cells and structures are required to maintain the electrolyte concentrations and homeostasis of the inner ear fluid (Koppl et al., 2018). Active transport mechanisms and essential structures for cation transport (e.g., Na^+^/K^+^-ATPase, Na^+^-K^+^-2Cl^–^ cotransporter and mitochondria) are needed to maintain a very high K^+^ concentration and recycling of electrolytes into endolymph (Wangemann, 1995; Ciuman, 2009). Vestibular and cochlear sensory hair cells, responsible for mechanosensation, are comparable in many respects including shape, morphology and the arrangement of stereocilia at the apical membrane.

However, a major physiological difference of both structures is the endolymphatic potential. The endolymphatic potential is a K^+^ equilibrium diffusion potential, in the cochlea across the apical membranes of the intermediate cells in the stria vascularis (Wangemann, 2002). Considering the inner ear as an electrical field, the endolymphatic potential is the quantity that determines the energy of charge. The endocochlear potential is as high as +80–100 mV (relative to perilymph) while the endovestibular potential is only up to + 10 mV. Mammals have an extremely high endocochlear potential compared to other animal species (Koppl et al., 2018). It is suggested that positive extracellular potentials around the hair cells augment the electrical gradient that is the major driving force for K^+^ and Ca^2+^ (calcium) influx during sensory transduction and subsequently enhance the neurotransmission of sounds (Hibino and Kurachi, 2006; Koppl et al., 2018). In contrast, the vestibular compartment demands lower endolymphatic potentials for its proper function, and equivalently has an endolymphatic potential of less than +10–15 mV among different species (fish, amphibia, reptiles, birds, and mammals) (Koppl et al., 2018). Lee and Jones (2018) demonstrated the functional discrepancy of both compartments after the disruption of K^+^ concentration causing acute reductions in the endolymphatic potentials in mice. While the cochlear response was significantly reduced as it requires a large transepithelial electrical potential for appropriate function, the vestibular response was unaffected as it is relatively insensitive to changes in the endolymphatic potentials.

**FIGURE 1:**
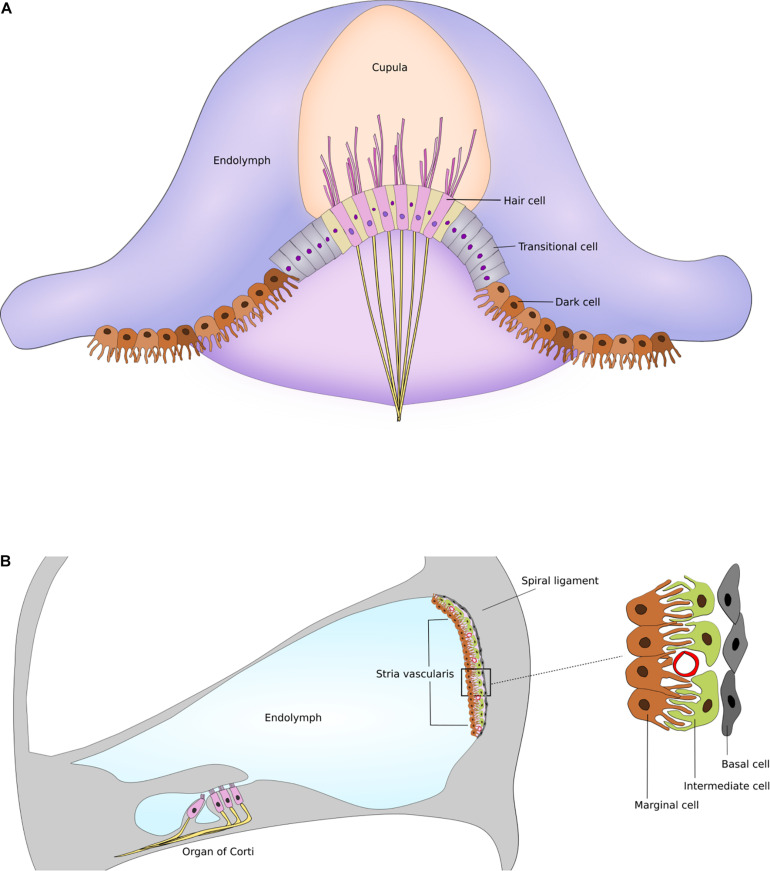
Supporting cells and structures of the inner ear. **(A)** Schematic drawing of dark cells and transitional cells in the semicircular canal. **(B)** Schematic drawing of stria vascularis within the cochlea illustrating the marginal, intermediate and basal cell layers. Figures are not drawn to scale.

The marginal cells of the cochlea, and the dark cells of the vestibule are responsible for endolymph production and homeostasis in maintaining the high K^+^ concentration (Takeuchi et al., 2000; Ciuman, 2009). Despite the morphological and functional similarities of both cells, the marginal cells are part of the multi-layered stria vascularis, while the vestibular dark cells form a single-layered epithelium (Ciuman, 2009). The complex structure of the stria vascularis at the lateral wall of the endolymphatic space of the cochlea - consisting of marginal, intermediate and basal cell layers—seems to underlie the high endocochlear potential. Intermediate cells and basal cells of the stria vascularis, and fibrocytes in the adjacent spiral ligament are responsible for the generation of the endocochlear potential (Takeuchi et al., 2000; Marcus et al., 2002). On the other hand, there is no analogous structure in the vestibular organ. Cochlear and vestibular structures and supporting cells are illustrated in Figure 1.

## Preclinical Data of Cisplatin Ototoxicity

Cisplatin is a cell-cycle non-specific agent that can kill a cell during any phase of the cell cycle, and so can act on both proliferating and non-proliferating cells (Mills et al., 2018). The neutral cisplatin is activated when it enters human cells, in that one or both of the chloride ions can rapidly be substituted by water (aquation) resulting in monovalent or divalent cations (Makovec, 2019).

The cytotoxic effects of cisplatin may occur via at least two major mechanisms. One mechanism is the formation of DNA (deoxyribonucleic acid) adducts, leading to DNA denaturation which blocks cell division processes (Makovec, 2019). Another mechanism of action involves the increased formation of reactive oxygen species and oxidative stress (Rybak et al., 2019). The intracellular pathway remains unclear but it seems to be related to apoptosis (programmed cell death) and necroptosis (programmed necrosis or inflammatory cell death) pathways (Callejo et al., 2015; Ruhl et al., 2019). These mechanisms ultimately lead to cell death via apoptosis (Mitra et al., 2017).

### Preclinical Data of Cochleotoxicity

**FIGURE 2:**
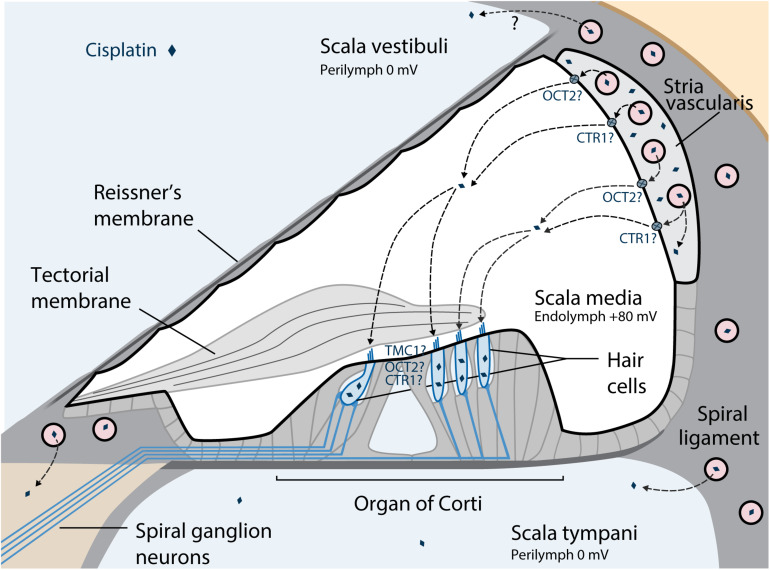
Suggested pathways of cisplatin trafficking in the cochlea. The major entry route for cisplatin entry into the cochlea is via the blood-strial barrier into the stria vascularis, and clearance into the endolymph from the stria vascularis prior to entry into hair cells across their apical membrane. Reproduced with modifications from Kros and Steyger (2018) with permission of Cold Spring Harbor Laboratory Press. OCT2, Organic cation transporter; CTR1, Copper transporter 1; TMC1, Transmembrane channel-like protein 1.

The suggested pathway of cisplatin trafficking from the bloodstream into the cochlea is displayed in Figure 2. To cause ototoxic damage, cisplatin must first cross the blood-labyrinth barrier, a specialized structure consisting of tight junction-coupled inner ear endothelial cells, which separates the inner ear tissues from the blood stream. The small size of the cisplatin molecule allows it to enter the inner ear via the blood-labyrinth barrier at the stria vascularis (Karasawa and Steyger, 2015; Chu et al., 2016; Breglio et al., 2017). Cisplatin then enters the endolymph in the scala media, potentially via the organic cation transporter 2 (OCT2) and/or copper transporter 1 (CTR1) in the marginal cells. Drug clearance from the stria vascularis into the endolymph seems to be a major contributing factor for ototoxicity among platinum-based chemotherapy (Ding et al., 2012; Gersten et al., 2020). From the endolymph, cisplatin can enter the cochlear hair cells via a variety of cation transporters, *via* the mechanoelectrical transduction channel pore, transmembrane channel-like protein 1 (TMC1), or by passive diffusion at apical membranes (Pabla et al., 2009; Ding et al., 2012; Waissbluth and Daniel, 2013; Kros and Steyger, 2018). Furthermore, platinum is retained in the human cochlea for many months to years after cisplatin treatment, while it is eliminated over the following days to weeks in other organs (Breglio et al., 2017). The hyper-deposition of cisplatin in the human cochlea appears to be a unique important mechanism of progressive and delayed-onset cisplatin-induced cochleotoxicity. It is suggested that cochlear cells are susceptible to cisplatin because of high drug uptake, high metabolic rate of these cells, and long-term retention of the drug (Schacht et al., 2012; Breglio et al., 2017).

The mechanisms of cisplatin-induced cochlear dysfunction are associated with cellular damage as well as disruption of cochlear homeostasis. After cisplatin administration, cellular degeneration was observed in outer hair cells of the organ of Corti, marginal cells of the stria vascularis, spiral ganglion cells, and synaptopathy between inner hair cells and spiral ganglion neurons (Brock et al., 2012; Chen et al., 2021).

Marginal cells of the stria vascularis could be the earliest targets of cisplatin ototoxicity (Thomas et al., 2006). Damage of the stria vascularis triggers functional alterations and apoptotic damage resulting in a reduced generation of the endocochlear potential and a disturbance in the electrolyte composition of endolymph, both of which are required for optimal auditory function (Laurell et al., 2007). A temporary reduction and recovery of endocochlear potential after cisplatin administration has been reported (Klis et al., 2002; Sluyter et al., 2003; Breglio et al., 2017). This occurrence could be primarily responsible for very early short-term reversible hearing loss after cisplatin exposure when a very slight degeneration of cochlear hair cells was reported (Tsukasaki et al., 2000; Klis et al., 2002). Cochlear function then recovers in parallel with the restoration of the endocochlear potential as long as the hair cells remain intact.

Cochlear outer hair cells showed the most prominent damage after cisplatin treatment, suggesting that they are more susceptible to injury and have a limited capacity for recovery (Callejo et al., 2015). Permanent hair cell loss is thought to be responsible for long-term irreversible hearing loss (Tsukasaki et al., 2000; Klis et al., 2002). Hair cell apoptosis could be caused by either direct injury after cisplatin entrance, or an indirect effect related to the disruption of cochlear fluid homeostasis.

### Preclinical Data of Vestibulotoxicity

There are fewer reports in the literature about cisplatin vestibulotoxicity than about cochleotoxicity. After topical cisplatin administration, degeneration of vestibular hair cells in utricular macula and cochlear outer hair cells was similar (Zhang et al., 2003; Cunningham and Brandon, 2006; Schmitt et al., 2009). Likewise, a parallel degree of vestibular and cochlear hair cells loss was reported after trans-tympanic cisplatin administration in the rat (Callejo et al., 2017). These results indicate that cisplatin, by local administration, seems to have a similar effect on hair cells of both compartments. However, the direct cisplatin exposure routes in these studies are not standard methods of cisplatin treatment in humans and so they lack the potential to evaluate physiological pharmacokinetics, especially vascularity and blood-labyrinth barrier function.

Conversely, minimal or no impact upon vestibular hair cells was reported after systemic cisplatin administration in guinea pigs, whereas there was a substantial loss of cochlear outer hair cells within the same setting (Schweitzer et al., 1986; Laurell and Bagger-Sjoback, 1991; Sergi et al., 2003). A minor loss of hair bundles after cisplatin exposure was also described (Nakayama et al., 1996; Ding et al., 1997). Overall, no study demonstrated extensive histological deterioration of vestibular organs after systemic cisplatin exposure as in analogous cochlear studies.

In animal models, some functional vestibular loss has been identified after cisplatin administration in semicircular canals (Sergi et al., 2003; Cheng et al., 2006; Takimoto et al., 2016), and otolith organs (Lo et al., 2015). The fact that morphological vestibular damage was not found at an early stage suggests that functional vestibular impairment may not be associated with sensory hair cell damage but other biochemical factors that cannot be seen in histological results (e.g., electrolyte or electropotential disturbance).

Currently, there is no study of cisplatin trafficking, uptake, and disruption of intracellular physiological pathways in the vestibular organs. It is possible that it involves the vestibular equivalent of the stria vascularis (dark cells and/or the transitional cells surrounding vestibular sensory epithelia, that are responsible for endolymph production and homeostasis) which has been demonstrated for aminoglycoside antibiotics (Liu et al., 2015; Kros and Steyger, 2018).

## Clinical Data of Cisplatin Ototoxicity

### Clinical Data of Cochleotoxicity

Hearing loss to variable degrees has been reported in 40–80% of patients treated with cisplatin depending on patient characteristics, drug dosage, and differences in tools and grading system (Landier et al., 2014; Frisina et al., 2016). The typical characteristics of cisplatin-induced hearing loss are irreversible, bilateral symmetrical sensorineural hearing loss affecting higher frequencies initially, then followed by lower frequencies (Rybak et al., 2019). Hearing loss often occurs in a dose-related and cumulative fashion (Landier, 2016; Paken et al., 2016). Forty percent of testicular cancer survivors who received cisplatin also complained of tinnitus which was significantly correlated with reduced hearing (Frisina et al., 2016). Therefore, monitoring of cochleotoxic effects of cisplatin is advised and implemented in clinical practice (Brooks and Knight, 2017; Clemens et al., 2019; Sprouse and Gozdecki, 2019). Age- and sex-adjusted audiometry is also suggested in long-term follow-up of adult cancer survivors to minimize the effects of age-related hearing loss (Skalleberg et al., 2020).

The phenomenon of cochlear dead regions involves loss of inner hair cells whilst outer hair cells are intact, can lead to difficulty understanding speech in noisy environments. This situation has been reported in a small cohort of adults who had undergone cisplatin chemotherapy (Schultz et al., 2019) but this result has not yet been corroborated by other studies.

### Clinical Data of Vestibulotoxicity

The clinical evidence regarding the potential of cisplatin vestibulotoxicity is limited in the published literature. The reported rate of abnormal caloric or rotational tests associated with cisplatin varied considerably from 0 to 50% (Prayuenyong et al., 2018). Some limitations of published studies include small numbers of patients, and different methods of evaluation and of criteria for abnormality.

Recently, we reported that all of a group of 65 adult survivors of cancer who had completed cisplatin treatment had normal video Head Impulse Test (vHIT) results (Prayuenyong et al., 2021). On the other hand, 14% of patients complained of hearing change after cisplatin treatment, 44% had new-onset or worsening tinnitus, and 29% had abnormal audiogram results which are in line with current literature in cancer survivors receiving standard dosage of cisplatin (100–400 mg/m^2^). The normal vHIT results indicate that the vestibulo-ocular reflex (VOR) activity is unaffected after cisplatin treatment. No evidence of corrective saccades was found, indicating that there was no subclinical vestibular impairment. The vHIT has the major advantage of specificity but at the expense of sensitivity (Halmagyi et al., 2017). This means that a positive finding of abnormality on vHIT can strongly rule in vestibular disorder, but that a negative result may not be relied upon to exclude vestibular pathology. However, the vHIT assesses VOR function in the high frequency where it is physiologically most relevant. Additionally, benign paroxysmal positional vertigo (BPPV) was relatively prevalent in this group of patients (9.2%). This figure is higher than the life-time prevalence of BPPV (2%) (von Brevern et al., 2007), and vertigo (7%) (Neuhauser et al., 2005) in the adult general population.

To date, there is no study of patients treated with cisplatin that incorporates the caloric test, rotational chair test, and vHIT to evaluate the VOR across different frequency ranges in the same setting. In case of aminoglycoside ototoxicity, vestibular function in low-mid frequency ranges was selectively affected but high frequency function was spared (Walther, 2017). It is thus possible that the caloric and rotational chair tests are more sensitive tools to detect vestibular impairment after ototoxic medication treatment (American Academy of Audiology, 2009).

## Proposed Hypothesis of Preferential Cochleotoxicity of Cisplatin

In general, cisplatin affects both cochlear structure and function, yet has a much lower likelihood to do so in the vestibular organ. One plausible explanation is that cisplatin does affect vestibular end organs, yet only a relatively low occurrence of vestibular symptoms is reported due to bilateral effects, insidious onset, and effective compensation mechanisms (Lacour et al., 2016). However, the current evidence does not support this notion and rather suggests that cisplatin does not cause vestibular insult or does so to a very limited extent. This raises the question why the cochlea is more vulnerable while the vestibule is generally preserved after cisplatin treatment.

The observations regarding morphological, biochemical, and functional changes after cisplatin exposure might shed some lights on the mechanisms of ototoxicity. The distinct patterns of hair cell loss after topical and systemic cisplatin administration could be explained by different drug trafficking across the blood-labyrinth-barrier or different degrees of cisplatin entry into sensory hair cells in the cochlear and vestibular compartments. Different endolymphatic potential status and supporting structures of the cochlear and the vestibular labyrinth might explain this phenomenon. The high electrochemical driving force of the endocochlear potential at 80–100 mV might strongly drive cisplatin, provided it is in its aquated, positively charged forms, to enter the hair cells (Koppl et al., 2018; Kros and Steyger, 2018). This is boosted by the −40 to −70 mV resting membrane potential of cochlear hair cells to generate a substantial electrical gradient of 120–170 mV across the apical membrane of the hair cells (Kros and Steyger, 2018). The vestibular endolymph has a smaller endolymphatic potential of 0–10 mV; thus, cisplatin might be less likely to enter vestibular hair cells (Koppl et al., 2018). Note, however, that cisplatin is predominantly neutral in a chloride-rich solution (which includes endolymph) and there is no study of cisplatin trafficking and concentration in the inner ear to support this assumption. Further studies are needed to tackle the issue of drug trafficking and uptake in the vestibular organs. Better understanding of the stria vascularis might shed further insight into the ototoxic mechanisms of cisplatin and otoprotective strategies to preserve hearing during systemic cisplatin treatment.

Cisplatin initially damages the outer hair cells at the basal turn of the cochlea, resulting in hearing loss at higher frequencies. There are at least two possible explanations for the sensitivity to cochleotoxic damage of cisplatin along the cochlear spiral. The first one relates to different drug distribution along the base-to-apex in the cochlea that facilitates greater cisplatin uptake in the basal part. Cisplatin signal intensity was highest in the cochlear base, and it generally decreased with progression toward the apex (Breglio et al., 2017). The concentration of cisplatin in scala tympani perilymph was fourfold higher in the basal turn of the cochlea than in the apex at 10 min after the administration (Hellberg et al., 2013). A second explanation concerns a greater intrinsic susceptibility of basal cochlear hair cells to cisplatin. Sha et al. (2001) showed that the base-to-apex vulnerability of hair cells remained when all parts of *ex vivo*, organotypic cultures of the cochlea were exposed to cisplatin. Also, they found a significant lower level of the antioxidant glutathione in basal outer hair cells compared with apical outer hair cells, suggesting that basal outer hair cells are more vulnerable to free-radical damage than apical ones. On the other hand, the preferential frequency of cisplatin vestibulotoxicity is unclear. It is possible that vestibular impairment is not homogenous across the frequency range. Unlike the cochlea, which has a well-structured tonotopic arrangement, the motion- and vibration-sensitive arrangement of the vestibular organs is not well structured.

A recent finding of the relatively high prevalence of BPPV after cisplatin treatment is of interest in this regard (Prayuenyong et al., 2021). The pathophysiology of BPPV involves displaced otoconia (extracellular calcium crystalline structures) from the utricular macula into the semicircular canal (Lee and Kim, 2010). It is associated with biochemical disruption of inner ear fluid, particularly Ca^2+^ metabolism, which is not necessarily correlated with hair cell injury. Cisplatin could cause electrochemical alteration of Ca^2+^ homeostasis in the vestibular compartment rather than hair cell damage (Scott et al., 1995). The reduction of Ca^2+^ concentration in endolymph induces the release of Ca^2+^ from otoconia and otoconial detachment from the otolithic membranes. Alternatively, limited physical activities due to general fatigue of cancer patients could also underlie the relatively high rate of BPPV in this sample (Pollak et al., 2011). These observations warrant further investigation, especially the effect of cisplatin in disrupting ionic hemostasis in the inner ear.

## Conclusion

In general, cisplatin ototoxicity appears to target cochlear structures resulting in hearing loss and/or tinnitus. Definite vestibular dysfunction after cisplatin treatment has not been consistently reported in the current literature. Cisplatin might have different pattern of drug trafficking across the blood-labyrinth-barrier or varying degrees of entry into hair cells in the cochlear and vestibular compartments. The endocochlear potential might also increase uptake of aquated cisplatin into the cochlear hair cells through cation transporters or the mechanoelectrical transduction channels. Although the VOR was generally unaffected, other vestibular effects of cisplatin such as biochemical disruption are possible. Further investigations are warranted for greater insight into the mechanisms of cisplatin trafficking, cellular uptake kinetics, and electrochemical disruptions. Better understanding of the stria vascularis might shed new light on ototoxic mechanisms and inform the development of otoprotective interventions to moderate cisplatin-induced ototoxicity.

## Author Contributions

PP wrote, revised, and edited the manuscript. DB wrote, reviewed, revised, and edited the manuscript. CK and PS reviewed and edited the manuscript. All authors contributed to the article and approved the submitted version.

## Conflict of Interest

The authors declare that the research was conducted in the absence of any commercial or financial relationships that could be construed as a potential conflict of interest.

## Publisher’s Note

All claims expressed in this article are solely those of the authors and do not necessarily represent those of their affiliated organizations, or those of the publisher, the editors and the reviewers. Any product that may be evaluated in this article, or claim that may be made by its manufacturer, is not guaranteed or endorsed by the publisher.
